# Stem cell sheet interpositioned between the tendon and bone would be better for healing than stem cell sheet overlaid above the tendon-to-bone junction in rotator cuff repair of rats

**DOI:** 10.1371/journal.pone.0266030

**Published:** 2022-03-24

**Authors:** Jae hee Choi, In Kyong Shim, Myung Jin Shin, Yu Na Lee, Kyoung Hwan Koh

**Affiliations:** 1 Asan Institute for Life Sciences, Asan Medical Center, University of Ulsan College of Medicine, Seoul, Korea; 2 Himchan Hospital Bupyeong, Incheon, Korea; 3 Department of Orthopaedic Surgery, Asan Medical Center, University of Ulsan College of Medicine, Seoul, Korea; Universita degli Studi di Perugia, ITALY

## Abstract

**Background:**

Although stem cells might enhance natural enthesis healing in surgical rotator cuff repair, not much attention has been given to the delivery and location of delivering stem cells. The purpose of this study to know where to locate those stem cells during repair.

**Methods:**

Animal model of chronic rotator cuff tear was created in 24 rats. Adipose-derived stem cells were engineered as a sheet and transplanted 1) between a torn tendon and humerus (interposition group) or 2) over a repaired tendon-to-bone junction (overlay group) at the time of surgical repair. Tracking of stem cells with overexpression of green fluorescent protein (GFP) were carried out at the time of sacrifice in additional 4 shoulders in each group. Histological and Biomechanical evaluation was performed to compare the differences in tendon-to-bone healing.

**Results:**

Histology showed increased fibrocartilage, a clear boundary at the mineralized fibrocartilage, abundant collagen type III, and higher total scores, especially in the interposition group. GFP-overexpression was observed at the transplanted site at 2 weeks after repair. Although two groups where stem cell sheets applied showed higher load to failure than the repair-only group, the load to failure was not different between the interposition and overlay group.

**Conclusion:**

In the chronic rotator cuff repair model, stem cell sheets enhanced regeneration of the tendon-to-bone junction. This regeneration was effective when the stem cell sheet was interpositioned at the tendon-to-bone interface.

**Level of evidence:**

Basic Science Study; In Vivo Animal Model; Histology and Biomechanics.

## Introduction

Despite the continued development of surgical treatment, the risk of retear still exists and remains a clinically essential issue in the surgical treatment of rotator cuff tears [1, 2]. Many studies have reported surgical techniques, fixation methods, and implant advancement to prevent retears after rotator cuff repair and improve structural properties [3–6]. Nevertheless, high retear rates are still reported in large-to-massive tears, and several biological studies have been conducted to reduce the retear rate [7, 8]. Recently, as methods for biological healing are enhanced, stem cell-based treatment has attracted attention in improving the regeneration potential of tendon [9–13]. Notably, mesenchymal stem cells (MSCs) are widely used in tissue engineering because they are easy to separate and culture from various tissues. Indeed, MSCs have been trialed variably as effective treatment methods for cell-based tendon regeneration research. However, no clear effect has been demonstrated so far, and the success at the cellular level is limited for application in the preclinical or clinical stage.

Regarding delivery, stem cells can be applied to lesions in various ways, such as injecting cells or culture medium of the cells, to induce tissue regeneration [10, 12, 14]. However, this method is challenging to apply in a specific area and maintain for a long time. Survival at the repair site where the MSCs were applied with a single injection is frequently doubtful, and the MSCs can wash out from the lesion. Moreover, we cannot precisely determine whether the applied stem cells directly differentiate into a tendon-to-bone junction tissue or act with its paracrine effect. Thus, it is unclear whether those stem cells should be applied between the torn stump of the cuff tendon and greater tuberosity right before repair completion, or the stem cells should be applied over the repair site right after the repair.

Engineered stem cells in a cell sheet form can easily adhere to tissues and continuously function at the application site [15, 16]. Recent studies have demonstrated that a stem cell sheet could be effectively applied to a surgical site as a stem cell delivery tool [17]. A logical subsequent step was a study to determine optimal locations and methods for using these stem cell sheets. This study verifies if the sheet form of MSCs in rotator cuff repair is an effective way of enhancing healing and to know where to locate those stem cells during repair.

Therefore, we hypothesized that biological augmentation for healing would be more effective when the stem cell sheet was located explicitly between the torn tendon and enthesis than over the repair site.

## Materials and methods

This study was approved by the Institutional Animal Care and Use Committee (IACUC of Asan Institute for Life Sciences, 2019-02-059 & 2019-02-183) and was conducted in compliance with the ARRIVE guidelines.

### Isolation and culture of primary adipose-derived stem cells from Sprague–Dawley rat inguinal fats

Rat adipose-derived stem cells (ASCs) were isolated from the inguinal fat tissue of Sprague–Dawley (SD) rats aged eight weeks (Orient Bio, South Korea). The stem cells were isolated from one normal rat to make sheets for transplantation using a previously published method [15]. To track the location of the stem cells after transplantation, green fluorescent protein (GFP)-expressing ASCs were isolated from a SD-GFP-transgenic rat (Japan SLC Inc., Hamamatsu, Japan) and used to produce cell sheets. The inguinal fat tissue harvested from the rats was digested using 0.1% (w/v) collagenase type I (Worthington, USA) dissolved in a warm phosphate-buffered saline (PBS) (Welgene Inc., Gywongsan, South Korea) for 1 h at 37°C. The isolated rat ASCs were cultured in Dulbecco’s modified Eagle medium (DMEM) low-glucose medium (Gibco, Thermo Fisher Scientific, USA) containing 10% (v/v) fetal bovine serum (FBS) and 1% antibiotic–antimycotic (A–A) solution in an incubator (37°C and 5% CO₂). After incubation for 24 h, the medium was changed to fresh culture medium. The cells were cultured up to passage 3.

### Characterization of rat ASCs

#### Flow cytometry analysis using surface makers

To analyze MSC surface markers, rat ASCs were seeded at passage 3 in 6-well plates (Nunc, Roskilde, Denmark). After 24 h, the cells were incubated with antibodies for 1 h at 4°C in darkness and washed twice using PBS. Flow cytometry (CanDo, BD Biosciences, California, USA) analysis was performed, and the following antibodies were used: PE-isotype, PE-CD29, PE-CD31, PE-CD45, FITC-isotype, FITC-CD73 (BD, USA), FITC-CD90, and MHC class I (AbD serotec, Bio-Rad Laboratories, California, USA). This analysis was performed in triplicate.

#### Confirmation of differentiation potential

For adipogenic differentiation, the cells were incubated in an adipogenic medium (Gibco, Life Technologies, USA) for two weeks. The cells were then stained with Oil Red O solution (Sigma-Aldrich, USA) for 5 min. For osteogenic differentiation, the cells were incubated in a DMEM–low glucose medium containing 1% A–A, 10-nM dexamethasone, 10-mM β-glycerophosphate, and 50-μM ascorbic acid. After four weeks, the cells were stained with 2% (w/v) Alizarin Red solution (Sigma-Aldrich, USA) for 30 min. Next, the cells were seeded at 1 × 10^6^ cells/mold (StemFIT3D; MicroFIT, Seongnam, Korea) in a normal culture medium to generate a spheroid form for chondrogenic differentiation. After 24 h, the cells in the spheroid form were transferred to a 100-mm cell culture dish and incubated in a chondrogenic medium (Gibco, Life Technologies, USA) for 2 weeks. The medium was changed every 3 d. Then, the cells were stained using Alcian Blue (Sigma-Aldrich, USA) for 30 min while maintaining the spheroid form.

#### Immunocytochemistry

Rat ASCs were seeded at 1 × 10^5^ cells/well in 24-well plates for 24 h. The cells were incubated in an advanced DMEM/F12 medium containing 2% FBS, 100-ng/ml GDF-7 (R and D system, MN), and 50-μg/ml ascorbic acid (Sigma-Aldrich, USA), and the medium was changed every 3 d. After 2 weeks, the cells were fixed using 4% paraformaldehyde (Invitrogen, USA) for 30 min and blocked using 3% bovine serum albumin (Cellnest, New Jersey, USA) for 1 h. Fixed cells were incubated with tendon-related antibodies, scleraxis, and tenomodulin (Abcam PLC, Cambridge, UK) overnight at 4°C with a 1:500 dilution ratio. Then, the cells were washed twice and incubated with a green fluorescent conjugated secondary antibody for 90 min. Nucleic acids were stained with Hochest 33342 (Invitrogen, California, USA) for 5 min. In the same manner as the cells, the prepared rat ASC-derived cell sheets were stained after the induction of differentiation using GDF-7 (n = 3).

#### Quantification of genes by reverse transcription-polymerase chain reaction

The rat ASCs were seeded at 2 × 10^5^ cells/well in 6-well plates for 24 h. The cells were incubated with GDF-7 in the same conditions as those used for immunocytochemistry. After two weeks, the treated cells were harvested, and total RNA was extracted using TRIzol reagent (Thermo Fisher Scientific, Massachusetts, USA). A quantitative reverse transcription-polymerase chain reaction (RT-qPCR) was conducted using SYBR Green PCR Master Mix (Applied Biosystems, California, USA). Primers were prepared using sequences verified in previously published studies [18, 19]; the sequences are summarized in Table 1. *GAPDH* was used for normalization. The gene expression levels were measured using an ABI PRISM 7900HT (Applied Biosystems, California, USA) PCR machine, and the relative gene expression levels were quantified using 2^−ΔΔCt^ values (n = 3).

**Table 1:** Primer sequence information. The sequences below were identified in NCBI’s Primer-BLAST.

Primer	GenBank file	Sequence (5′ → 3′)	
*Scleraxis*	NM_00130508.1	Forward	TGGCCTCCAGCTACATTTCT
		Reverse	TGTCACGGTCTTTGCTCAAC
*Tenomodulin*	NM_022290.1	Forward	GGGATTGACCAGAATGAGCAA
		Reverse	GGTGCGGCGGGTCTTC
*Tenascin C*	XM_008763758.2	Forward	CAGAAGCCTTGGCCATGTG
		Reverse	GCACTCTCTCCCCTGTGTAGGA
*Collagen Type Ⅲ*	NM_032085.1	Forward	TGATGGGATCCAATGAGGGAGA
		Reverse	GAGTCTCATGGCCTTGCGTGTTT
*Collagen Type Ⅰ*	NM_053304.1	Forward	GCCAAGAAGACATCCCTGAA
		Reverse	GCAGAAAGGACAGCACTCGC
*GAPDH*	XM_017592435.1	Forward	TCTCTGCTCCTCCCTGTTCTA
		Reverse	ATGAAGGGGTCGTTGATGGC

### Cell sheet fabrication

**Fig 1:**
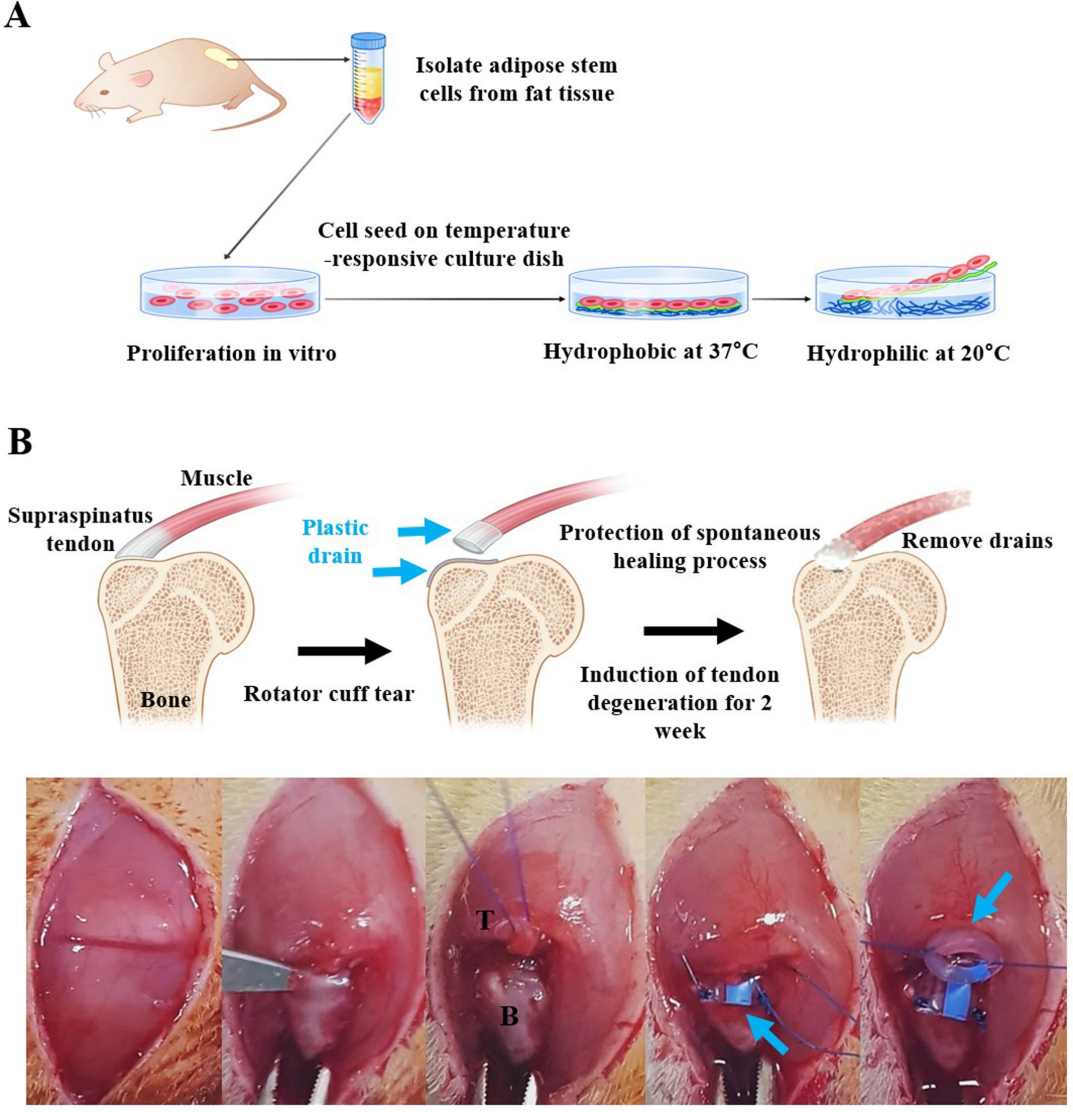
Schematic illustrations of the cell sheets for enhancing regeneration after rotator cuff tear repair. (A) The concept of producing a cell sheet using adipose-derived stem cells isolated from rats. The sheets were harvested by changing the hydrophilicity of the dish surface and decreasing the temperature. (B) The process of creating a chronic rotator cuff tear model. The tendons were separated entirely and maintained for two weeks using a plastic drain in two places to block spontaneous healing. T: tendon, B: bone, Blue arrow: plastic drain.

Rat ASCs were seeded at passage 3 at 1.2 × 10^6^ cells/dish in a temperature-responsive dish (35 mm; Thermo Fisher Scientific, Massachusetts, USA) and incubated for 24 h at 37°C. When the temperature of the dish is lowered, the attached cells are detached from the surface of the dish. Using this principle, cells were obtained in the form of sheets. First, the cell sheets were cultured in a normal culture medium at 37°C to focus on engineered stem cell sheets for the tendon-to-bone healing. Then, the cells were removed from the dish by changing to a lower temperature of 20°C using cold medium (Fig 1A). The removed cells shrunk to form a small round sheet [16]. One round sheet per shoulder was used in the sheet transplant group.

### Animal model of chronic cuff tear and application of stem cell sheet

Twelve eight-week-old male SD rats with a weight of 250 g (24 shoulders) were used for this study. Before surgical treatment, 0.3 cc of a mixed solution containing 50-mg/kg tiletamine/zolazepam (Virbac, Carros, France) and 10-mg/kg xylazine (Bayer HealthCare, Leverkusen, Germany) was intramuscularly (IM) injected for anesthesia. The first step was making a tear of the supraspinatus tendon (SSP). SSP attached to the great tuberosity (GT) of the humerus was cut as close to the footprint as possible. Then, a plastic drain was used to block the spontaneous healing in both the torn tendon stump and GT of the humerus. Notably, a stump on the torn tendon side was sealed with the plastic drain using a 5–0 Ethibond suture (Somerville, USA) to block spontaneous healing (Fig 1B). After making a tear on the SSP, the rats were maintained with free cage activity for two weeks. Thus, the rat model of chronic tear was completed [20, 21]. For preventing infection and pain relief, 50-mg/kg ampicillin and 3-mg/kg ketorolac, respectively, were injected IM every day for three days after the tear model was created. The models of chronic rotator cuff tears were made identically in all rats.

Two weeks after making a tear model, surgical repair was performed. Same method of anesthesia with 0.3 cc of a mixed solution containing 50-mg/kg tiletamine/zolazepam (Virbac, Carros, France) and 10-mg/kg xylazine (Bayer HealthCare, Leverkusen, Germany) via intramuscular (IM) injection was used for repair as well. Since this study aimed to see where we should locate stem cells for biological augmentation during the repair, rats were allocated to one of three groups. As a control, surgical repair was performed alone in four rats (repair-only group; eight shoulders) without applying a stem cell sheet. To repair a torn tendon, GT of the humerus head was drilled using a 0.5-mm drill, and the SSP was transosseously sutured using a 5–0 Ethibond suture [22, 23]. The experimental group was divided into two groups according to the location of the stem cell sheet transplantation. In the first experimental group (overlay group; 8 shoulders), the repair was conducted in the same manner as in the control group. Then, the cell sheet was attached above the repair site so that it covered the area from a part of the humerus to the tendon across the repair site. Additionally, the prepared rat ASC sheet was transferred using a thin membrane shifter (Thermo Fisher Scientific, Massachusetts, USA). First, the medium in the dish with the cell sheet was removed. Next, the shifter was placed on the cell sheet; therefore, the sheet was attached to the shifter and then transferred from the dish to the tissue. After approximately 1 min, it was confirmed that the cell sheet was attached entirely, and then the shifter was removed.

In the second experimental group (interposition group; 8 shoulders), a hole was drilled on the GT identically. A 5–0 Ethibond suture was passed through the drill hole and the torn tendon to prepare a repair. The stem cell sheet was transplanted between the tendon and bone (the GT of the humerus), and the shifter was removed after confirming that the cell sheet was attached. The repair was completed by pulling and knot-tying the 5–0 Ethibond passed through the bone and torn tendon. Ampicillin (50 mg/kg) and ketorolac (3 mg/kg) were injected once daily for preventing infection and postoperative pain until the third day after surgery.

**Fig 2:**
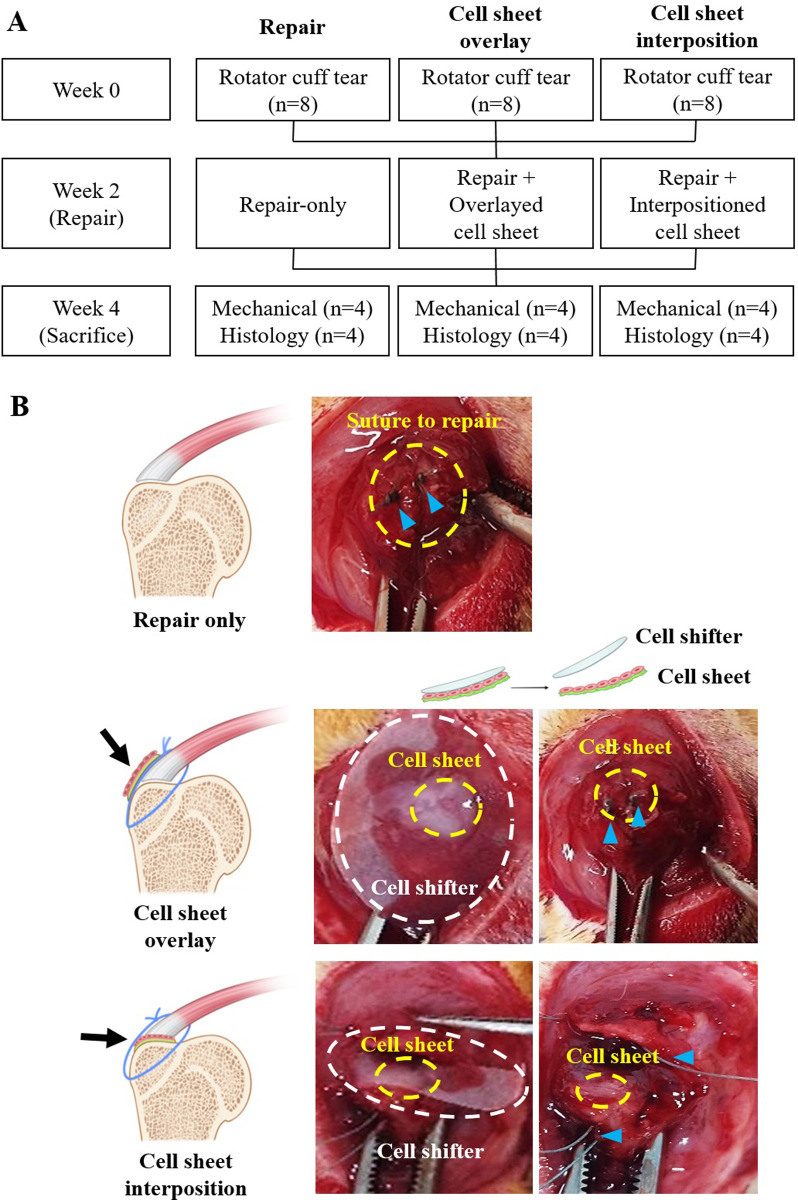
Study design for experiments using rats. (A) The experiment was divided into three groups, with eight rats in each group. (B) The completion of surgical repair and stem cell sheet transplantation. Control group: a surgical repair-only, experimental group: Cell sheet transplantation on top of the repair site (overlay group) or between the tendon and bone (interposition group). The cell sheet is highlighted with a yellow circle in the illustration, and the suturing thread is shown as a blue triangle in the images.

After two weeks of repair, the repair quality was analyzed after the sacrifice of all animals (Fig 2). All rats were humanely sacrificed via carbon dioxide asphyxiation for all evaluations.

### Evaluation

#### Histological estimation

After two weeks of repair, all animals in this study were sacrificed using carbon dioxide gas. Based on the tendon-to-bone junction on both shoulders, tissues were removed entirely, except for the humerus head and tendon part. We obtained 4-μm thick tissue sections through the following series of processes: formalin fixation, paraffin embedding, deparaffinization, and dehydration using a changed alcohol concentration.

Immunohistochemistry staining was conducted using a GFP-antibody (Abcam PLC, Cambridge, UK) in additional 4 shoulders of each group. The primary antibody was diluted at a 1:500 ratio. The pathology department performed all procedures requiring 3,3′-diaminobenzidine (DAB) staining. First, the sectioned tissues were stained using hematoxylin and eosin (H&E) solution (Sigma-Aldrich, USA). To confirm the presence of collagen fibers, the sectioned tissues were stained using Masson, Safranin O (Polysciences, Inc., Warrington, USA) kits, and Sirius Red staining kit (Abcam, UK). Another immunohistochemical staining was conducted using the same antibody used for cell analysis, scleraxis and tenomodulin. Likewise, the tissues were blocked using 3% BSA and incubated with scleraxis and tenomodulin antibodies overnight at 4°C with a 1:500 dilution ratio. Then, the tissues were incubated with a secondary antibody and observed under a microscope. The four shoulders were used for each group, and all images were taken using an EVOS microscope (Thermo Fisher Scientific, Massachusetts, USA).

The fibrocartilage region was analyzed using H&E, Masson, Safranin O, and Sirius Red staining using the following four criteria: bone, tendon, mineralized fibrocartilage, and vascularity (Table 2). There was no standard guideline validating the histological scores used to assess tendon-to-bone healing. The modified scoring system used in the previous studies was adopted to evaluate the recovery observed in histology [24–27]. The results were scored by two independent observers, and finally, the average value was evaluated [23, 28]. The length of the tidemark at the tendon-to-bone junction was confirmed by H&E staining. Fibrocartilage formed after the repair was observed under a microscope with Safranin O staining. The length of the total fibrocartilage and the represented tidemark was measured, respectively, and the length of the tidemark with the total length was converted into a percentage [29–31]. The fibrocartilage formation area and length was quantitatively analyzed using ImageJ (National Institutes of Health, Maryland, USA) based on the surgical site [32].

**Table 2:** Histological scoring system at the tendon-to-bone location.

Categories		Scores
**Bone**	Abundant	2
**Bone formation**	Moderate	1
**Architecture**	Lack	0
**Tendon**	Orientation (+) and thickness (+)	2
**Fiber orientation**	Orientation (+) or thickness (+)	1
**Continuity**	Orientation (−) and Thickness (−)	0
**Tendon-to-bone junction**	Abundant (+) and tidemark (+)	2
**Fibrocartilage formation**	Abundant (+) or tidemark (+)	1
**Tide mark**	Abundant (−) and tidemark (−)	0
**Vascularity**	Abundant	2
**Vascular formation around the tendon**	Moderate	1
	Lack	0
**Total scores**		0–8

#### Biomechanical comparisons

A biomechanical test was conducted using a machine capable of measuring the uniaxial tensile stress test (ST-1001; Salt Co., Ltd, Incheon, Korea). Four shoulders were used for each group, and to evaluate the tendon-to-bone junction, surrounding tissues were removed as much as possible. Sandpaper was attached to the proximal humerus side and then fixed to the distal part of the machine to control the slip. The tendon side was not sutured, but 2 mm above the junction was fixed tightly with a wide clamp. A gradient tension of 1 mm/min was applied to measure the load to failure and stiffness [33, 34].

### Statistical analysis

Statistical analysis was conducted using GraphPad Prism v.5 (GraphPad Software, California, USA). A one-way analysis of variance and post hoc analysis was conducted to compare the groups, and errors were corrected using Tukey’s method. The data were presented as mean and standard deviation. *P* < 0.05 was considered statistically significant, and *P* < 0.05 was marked as *, and *P* < 0.001 was marked as ** on the graph.

## Results

### Properties of rat ASCs

**Fig 3:**
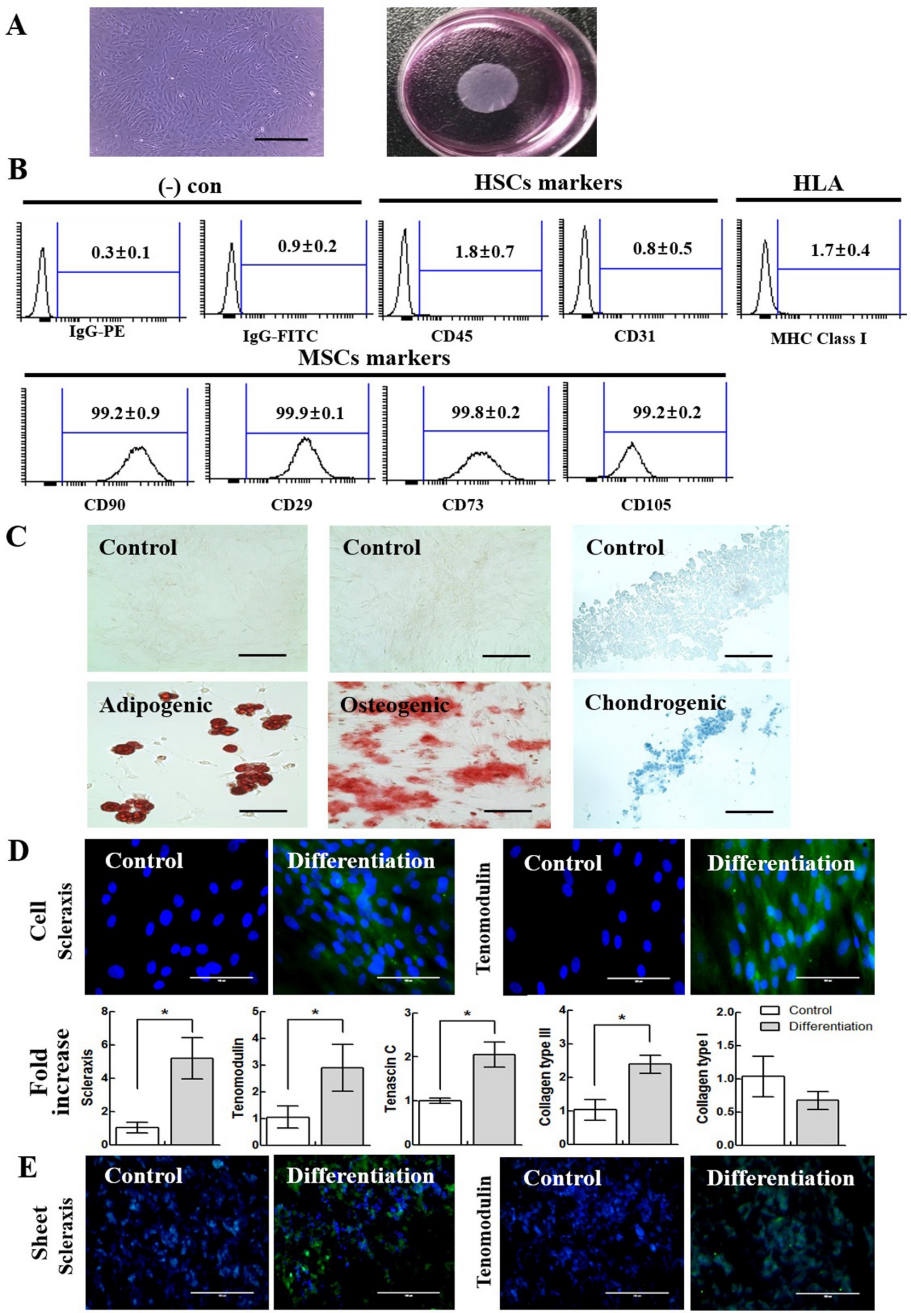
Characterization of rat adipose-derived stem cells (ASCs). (A) The morphology of the cell sheet with plastic adhesion (left) and the shape of the shrunken cell sheet (right). The size bar represents 400 μm (magnification 10X). (B) The expression of MSC markers in rat ASCs (n = 3). (C) The adipogenic, osteogenic, and chondrogenic differentiation potential of rat ASCs (n = 3). The differentiation sites are shown in red or blue. All size bars represent 100 μm (magnification 40X). (D) The tenogenic differentiation potential of rat ASCs with 100 ng/ml GDF-7 treatment for two weeks. The side that has not treated the GDF-7 was used as a control. The fluorescence images of tendon markers in rat ASCs (upper). All size bars represent 100 μm (magnification 40X). The gene expression level of representative tendon markers in cells. Error bars represent standard deviation. *P < 0.05 (n = 3) (lower). (E) The tenogenic differentiation potential of rat ASC-derived cell sheet with GDF-7 treatment for two weeks. Green: differentiated cells, blue: nuclei acid. All size bars represent 100 μm (magnification 40X).

In terms of cell morphology during the culture at passage 3 to make the ASC sheet, the cells were attached, and they appeared in a fibroblast-like morphology (Fig 3A, left). The rat ASC sheet was cultured to form a single layer in a circle (Fig 3A, right). The number of cells for the most robust cell sheet was optimized at 1.2 × 10^6^ cells/dish. The rat ASCs were 99.2% ± 0.9%, 99.9% ± 0.1%, 99.8% ± 0.2%, and 99.2% ± 0.2% positive for the MSC-specific surface markers CD90, CD29, CD73, and CD105, respectively. Moreover, ASCs did not express CD45, CD31 (hematopoietic stem cell), and MHC class I markers at 1.8% ± 0.7%, 0.8% ± 0.5%, and 1.7% ± 0.4%, respectively (Fig 3B). Adipogenic, osteogenic, and chondrogenic differentiation were confirmed by oil formation (Oil Red O staining), calcium accumulation (Alizarin Red), and aggrecan-rich extracellular matrix (Alcian Blue) (Fig 3C).

Since growth differentiation factor-7 (GDF-7) induces tenogenic differentiation [35–38], it was added to rat ASCs. As a result, GDF-7 treatment induced the overexpression of scleraxis and tenomodulin, representing tendon-specific gene markers. In immunocytochemistry, the differentiation was confirmed using fluorescence signals in the nuclear region and the cytoplasm (Fig 3D, upper) [39–43]. RT-qPCR showed that when the control values were normalized to 1 in each marker, the differentiation values were 5.18 ± 1.24, 2.9 ± 0.87, 2.05 ± 0.28, and 2.38 ± 0.27 for scleraxis, tenomodulin, tenascin C, and collagen type III, respectively. Thus, the tenogenic differentiation potential of rat ASCs was confirmed with an increase in the gene expression level (Fig 3D, lower). Additionally, the differentiation potential of GDF-7 was revealed in the cell sheet state. The overexpression of scleraxis and tenomodulin was confirmed in the cell sheet that induced differentiation (Fig 3E).

### Locations of transplanted rat ASC sheet *in vivo*

**Fig 4:**
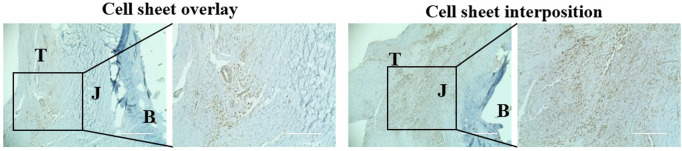
The confirmation of GFP expression depending on the location of the transplanted cell sheets. The cell sheets were transplanted above (left) or between (right) the tendon-to-bone junction. The GFP-tagging cells were used for the transplanted sheets, and it was confirmed two weeks after repair. GFP expression is located at the black box and shown as brown dots.

When the cell sheet remained at the application site and GFP was overexpressed, it was stained with brown dots, and its location was confirmed. In the overlay group, it was confirmed that the brown spots were distributed outside the junction. Alternatively, in the interposition group, GFP-positive cells were confirmed inside the junction. The rat ASC sheet was still present at the applied site two weeks after transplantation and could affect healing (Fig 4).

### Gross morphology and histology

**Fig 5:**
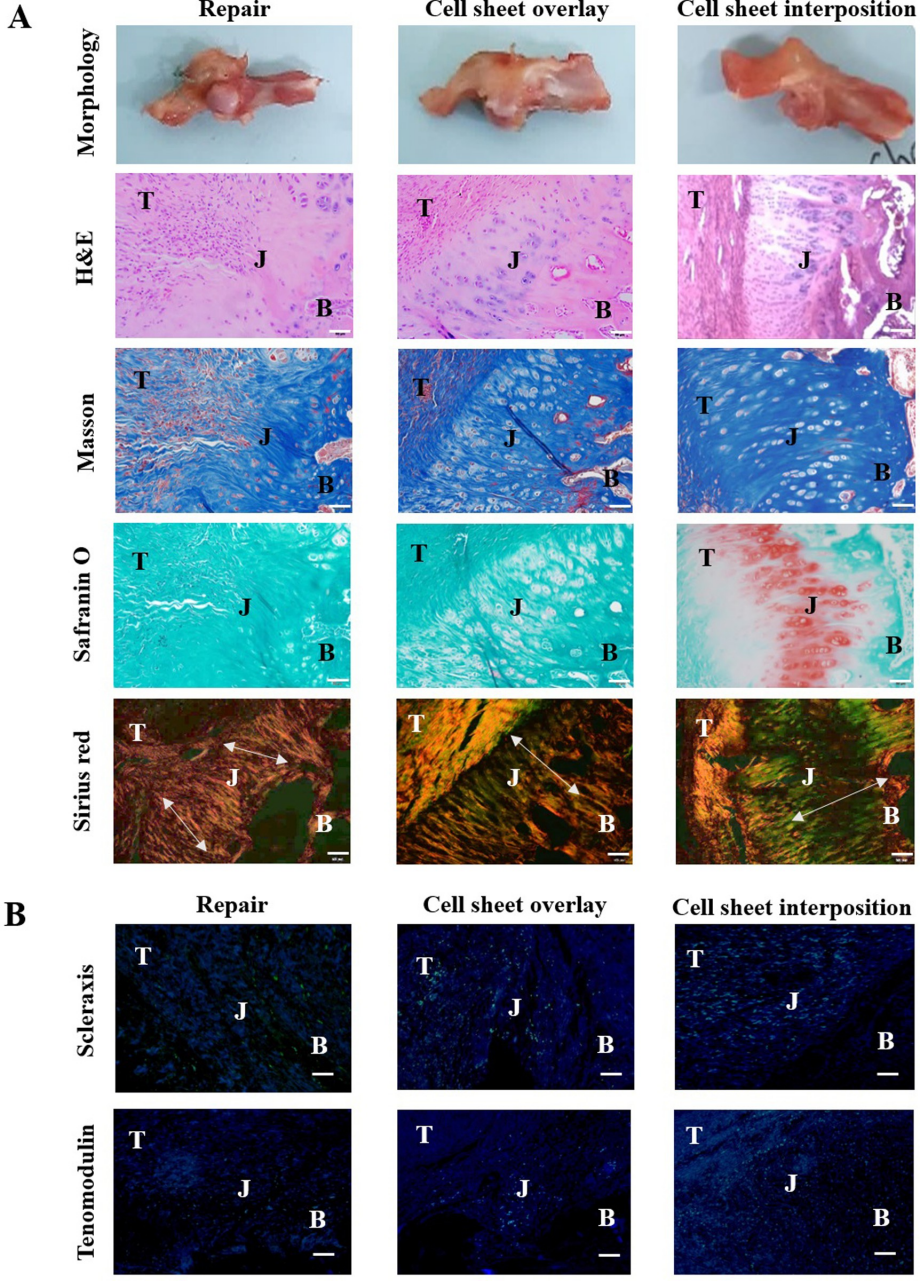
Histological analysis according to the application of the cell sheets after repair. (A) The different gross morphology depends on the location of the transplanted cell sheets and histological representation of the tendon-to-bone junction. T: tendon, J: junction, B: bone. The size bars represent 200 μm (magnification 20X). (B) The confirmation of the tendon marker expression for each group. The expression level of the markers was confirmed by green fluorescence. Blue dot is a nucleic acid. The size bars represent 200 μm (magnification 20X). All figures were selected as representative samples of each group.

After two weeks of repair, the overlay group had a higher amount of tissue in the region above the junction than the repair-only group. Furthermore, the interposition group demonstrated a tighter connected enthesis and a relatively thickened SSP tendon. In Masson’s trichrome (Masson) staining, a considerable amount of fibrocartilage was formed, and the boundary at the mineralized fibrocartilage became clear in the two groups transplanted with the stem cell sheet. In Sirius Red staining, the difference was confirmed that a collagen type III (yellow and green) was significantly more abundant in the group transplanted with the sheet, especially the interposition group. In scleraxis and tenomodulin immunostaining, the expression of the tendon markers was better in the cell sheet-transplanted groups than the repair-only group (Fig 5).

**Fig 6:**
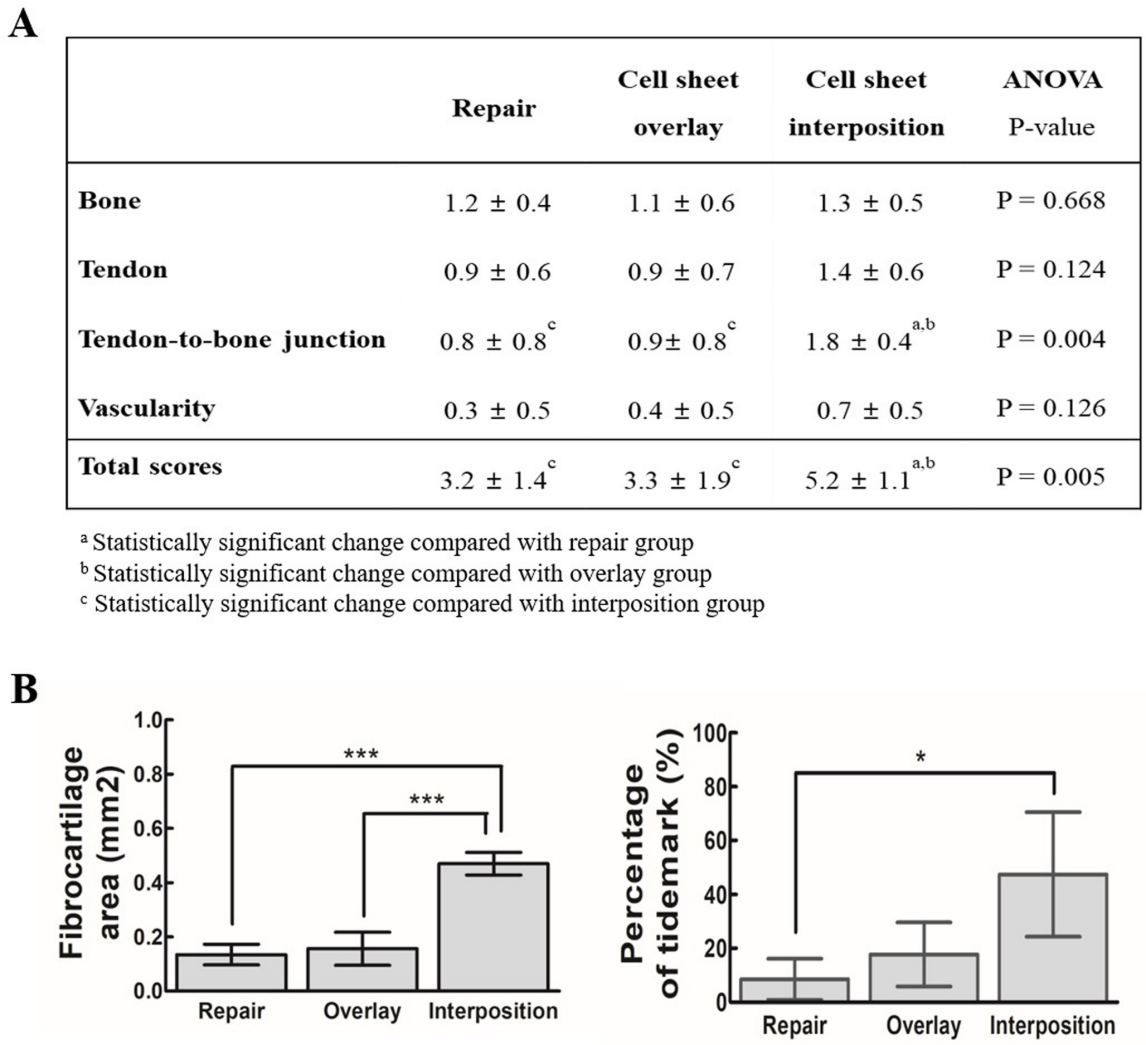
Histological scoring. (A) The three groups were evaluated on four criteria. The above subscript indicates statistically significant relationships between groups. (B) Graph quantifying (left) the relative fibrocartilage formation and (right) percentages of the produced tidemark length relative to the fibrocartilage width at the tendon-to-bone junction. Error bars represent standard deviation. *P < 0.05, ***P < 0.001 (n = 4).

On histological evaluation, the total score was significantly higher in the interposition group (5.2 ± 1.1) than those in the surgical repair-only (3.2 ± 1.4) and overlay (3.3 ± 1.9) groups (*p* = 0.005, Fig 6A). No bone formation or structural differences were observed in any of the three groups, and no statistically significant difference in the tendon and blood vessel formation was observed. However, a significant difference in the tendon-to-bone junction was found between the interposition and the other two groups (*P* = 0.004). In Safranin O staining, the area of the glycosaminoglycan region was quantified. Subsequently, there was a statistically significant difference between the interposition group (0.47 ± 0.04) and repair (0.13 ± 0.04) or overlay (0.18 ± 0.11) group (Fig 6(B), left) (*p* <0.001). Additionally, the length of the tidemark representing the boundary between non-mineralized and mineralized fibrocartilage at the tendon-to-bone junction was quantified to confirm the degree of recovery native-like enthesis. Subsequently, the length of the generated tidemarks was longer in the cell sheet-transplanted groups (overlay group: 17.73% ± 10.29%, interposition group: 47.41% ± 19.99%) compared with the repair-only group (8.50% ± 6.63%) (*p = 0*.*016*). Notably, it was significantly increased in the interposition group compared with the repair-only group (*p* = 0.019) (Fig 6B, right).

### Biomechanical test

**Fig 7:**
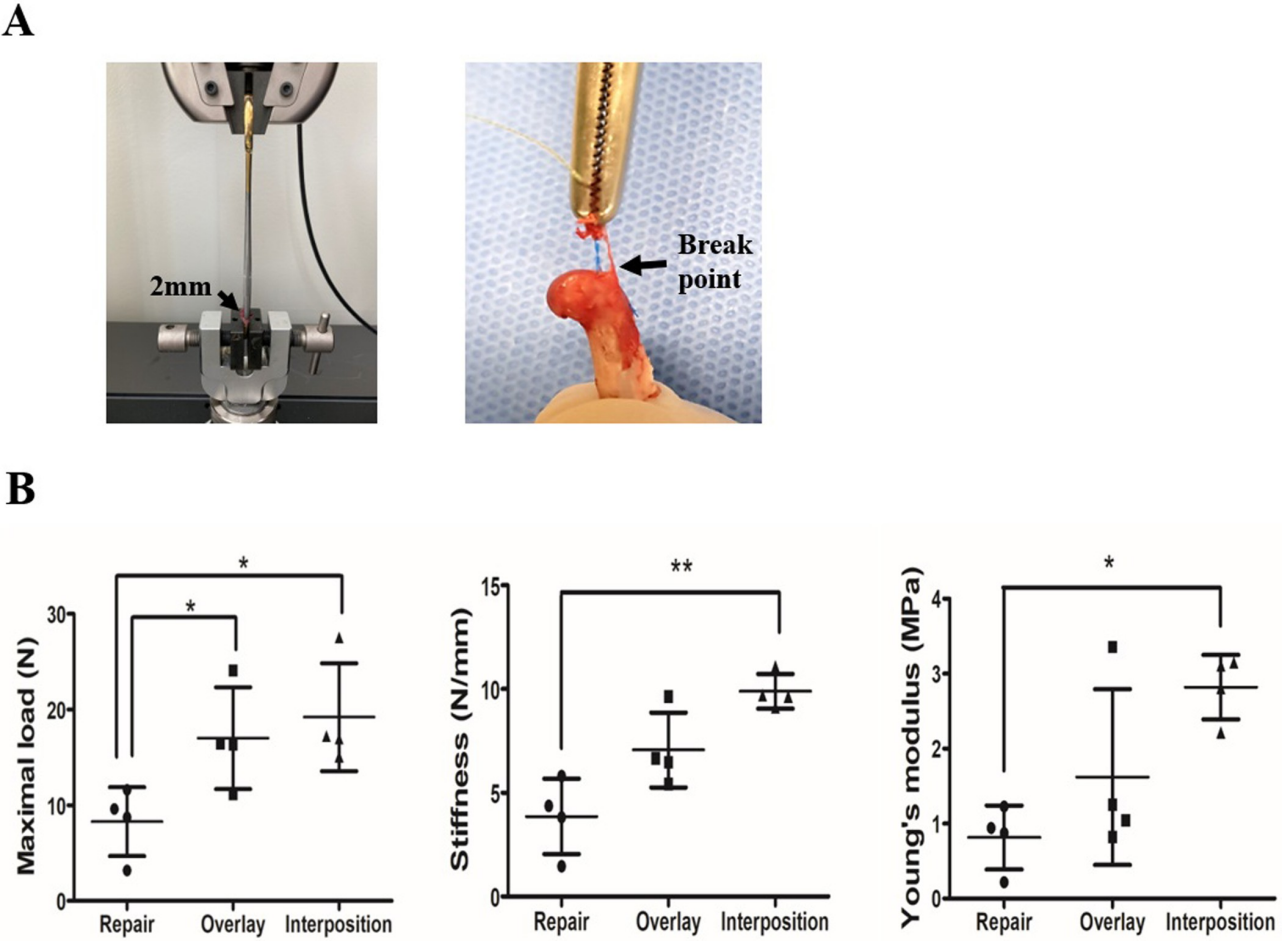
Biomechanical analysis of the application of cell sheets. (A) The image shows a tensile test device (left) and a mode of failure by tendon tearing at the enthesis (right). The 2 mm above from the junction was fixed, and a gradient tension of 1 mm/min was applied. (B) Three graphs indicate the maximal load, stiffness, and Young’s modulus of the tendon-to-bone interface. Error bars represent standard deviation. *P < 0.05, **P < 0.01 (n = 4).

Because of fixing 2 mm above from the junction and conducting a biomechanical analysis, it was confirmed that the breaking point was enthesis (Fig 7A). The maximum load to failure was significantly different between the three groups, especially between the surgical repair-only and overlay groups (8.29 ± 3.61 N and 17.02 ± 5.33 N, respectively) (*p = 0*.*035*), and between the surgical repair-only and interposition groups (8.29 ± 3.61 N and 19.20 ± 5.63 N, respectively) (*p = 0*.*017*). Regarding stiffness, the surgical repair-only group (3.86 ± 1.81 N/mm) was significantly different compared to the interposition group (9.89 ± 0.83 N/mm) (*p = 0*.*001*). However, in the overlay group (7.06 ± 1.80 N/mm), the numerical value was increased, but no statistically significant difference was observed. The Young’s modulus was significantly different between the repair group (0.82 ± 0.43 MPa) and the interposition group (2.82 ± 0.43 MPa) (*p* = 0.001), but the overlay group (1.62 ± 1.17 MPa) was not statistically differentiated (*p = 0*.*246*) (Fig 7B).

## Discussion

The prime purpose of this study was to determine how to deliver stem cells to the repair site effectively. Our study showed that the sheet form of stem cells was maintained at the application site for at least more than two weeks after the repair. Furthermore, stem cells applied between the torn tendon and GT were more effective in regeneration of tendon-to-bone interface than those placed above the repair.

Despite enormous advances in repair techniques and technologies, including instrumentation, retear remains a major concern after repairing the torn tendon. A reason for the retear following surgical repair could be a lack of healing process and regeneration. Initial continuity at the repair site provided by fibrous tissue should be replaced by scar-like unoriented tendon tissue, then remodeled to a tendon-to-bone interface with a mature mineralized fibrous cartilage layer. However, the lesions are limited in their self-regeneration ability due to the relatively low cell density, the distribution of blood vessels, and microenvironment changes. For this reason, surgical treatment is followed by slow regeneration and incomplete recovery of the biological properties and original functions [44–46]. Therefore, the biochemical and mechanical properties of the repair site never match with an original tendon-to-bone junction [47, 48].

This has prompted increased interest in biological approaches, and stem cells have inspired considerable attention over the past years. Besides the effect of stem cells on healing, delivery of the stem cells is a crucial issue in the outcome. However, not much attention has been given to this issue. Ideally, continuous and controlled delivery to maintain an effective concentration of stem cells should be provided. Proper localization of stem cells at the repair site should be ensured. A single injection of stem cells would be washed out and be ineffective, especially during the currently used arthroscopic rotator cuff repair environment. Recent arthroscopic surgeries are conducted in continuous saline irrigation. The sheet form of stem cells in our study would be a good option for continuous and controlled delivery of stem cells even in arthroscopic surgery. With a cell shifter without any special adhesion method, the transplantation of stem cells was not difficult in our study. The cells stayed at the application site and would play a role as a biological enhancement for at least two weeks after repair. It would be easy to apply even in the clinical setting. Our histological evaluation confirmed that those stem cells tagged with GFP remained even two weeks after application, whether transplanted over the repair site or between the tendon and bone (Fig 4).

Another issue in delivering stem cells in rotator cuff repair is where exactly the stem cells should be applied. For example, when we repair the torn tendon to the bone, it would be reattached on a remnant enthesis by apposition. Stem cells as a form of a sheet can be applied to the interface between the tendon and bone or over the whole repair site covering the surface from bone to tendon. Since the regeneration should occur at the entire contact surface between the tendon and enthesis, we can see that the cell sheet applied at the interface would have more benefit than overlaid application. Our study shows that the specific application site is another essential factor for efficient healing and the regeneration of the tendon-to-bone interface.

One characteristic histological finding of native enthesis at the tendon-to-bone interface that we attempted to recover is the formation of non-mineralized and mineralized fibrocartilage layers. We demonstrated that stem cell sheets enhanced regeneration with this native enthesis. When the stem cells were interpositioned between the tendon and bone, an increase in the fibrocartilage layer was statistically evident on histological exams. Even when the stem cells were overlaid on the adjacent part of the repair site, fibrocartilage formation was abundant compared to the surgical repair only; this result may be interpreted as the paracrine effect of stem cells. However, it could be observed in a more robust fibrocartilage layer and collagen alignment concerning interposition. This case can explain the direct differentiation potential of the stem cells and the possibility of the simultaneous paracrine effects.

This study had a limitation in that we could not evaluate the mechanism of the action of stem cells. Tracking of tenogenic, adipogenic, osteogenic, or chondrogenic differentiation of stem cells was impossible in the tendon-to-bone junction. Since retear occurs early after repair due to the insufficient healing process [49, 50], and the purpose of applying stem cells in our study was to enhance initial healing, we sacrificed the rats two weeks after repair for evaluation. It might have led to no biomechanical differences two weeks after repair in our study, and two weeks after repair might be too early to show biomechanical superiority of stem cells in a specific location of the application. There would be more biomechanical benefits with a longer duration after the repair and application of stem cells [17]. Additionally, although eight shoulders were compared per group, the sample size was relatively small for both histological and biomechanical tests. This might be another reason for no statistical differences in the biomechanical tests. However, despite the sample size limitations, we confirmed the apparent advantage in histological evaluation with this small sample. The results of our study might propose a feasible option for application as a preliminary study. For future clinical applications, intensive studies in large animals will also be required.

## Conclusion

In this study, stem cell sheets effectively enhanced the recovery process of enthesis (tendon-to-bone junction) in repairing chronic rotator cuff tears in a rat model. Notably, we could demonstrate that the recovery effect was further improved when the stem cell sheet was interpositioned between the tendon and bone.

## Supporting information

S1 Table The gene expression level of representative tendon markers in cells. Tenogenic differentiation potential of rat ASCs with 100ng/ml GDF-7 treated for two weeks.
(TIF)

S2 Table Histological scoring. The three groups were evaluated on four criteria.
(TIF)

S3 Table The relative fibrocartilage formation and percentages of the produced tidemark length relative to the fibrocartilage width at the tendon-to-bone junction. (TIF)

S4 Table Biomechanical analysis of the application of cell sheets. The maximal load, stiffness, and Young’s modulus of the tendon-to-bone interface.
(TIF)
