# Supplementary figures and images for: Stem cell sheet interpositioned between the tendon and bone would be better for healing than stem cell sheet overlaid above the tendon-to-bone junction in rotator cuff repair of rats
Source: PLoS One. 2022 Mar 24;17(3):e0266030. doi: 10.1371/journal.pone.0266030 (PMC8947210; doi:10.1371/journal.pone.0266030)

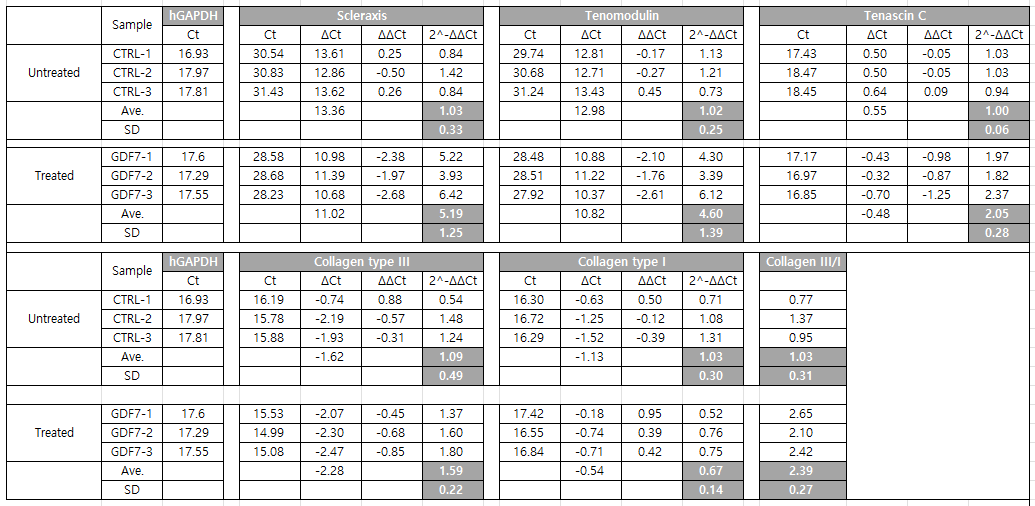

Supplement: S1 Table — Tenogenic differentiation potential of rat ASCs with 100ng/ml GDF-7 treated for two weeks. (TIF) [file pone.0266030.s001.tif]

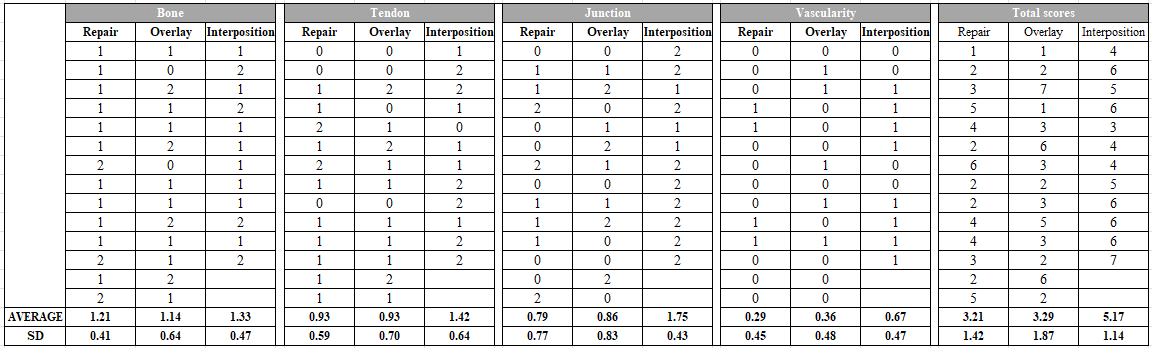

Supplement: S2 Table — The three groups were evaluated on four criteria. (TIF) [file pone.0266030.s002.tif]

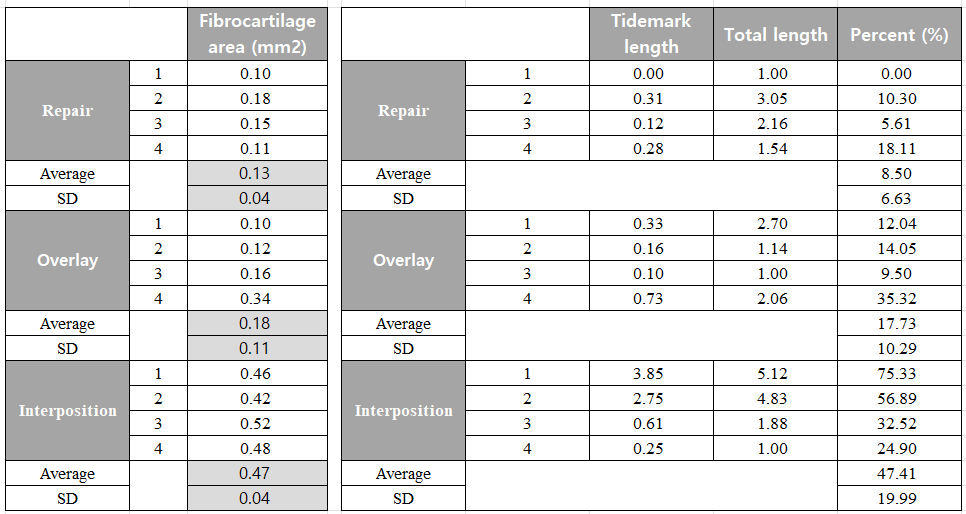

Supplement: S3 Table — (TIF) [file pone.0266030.s003.tif]

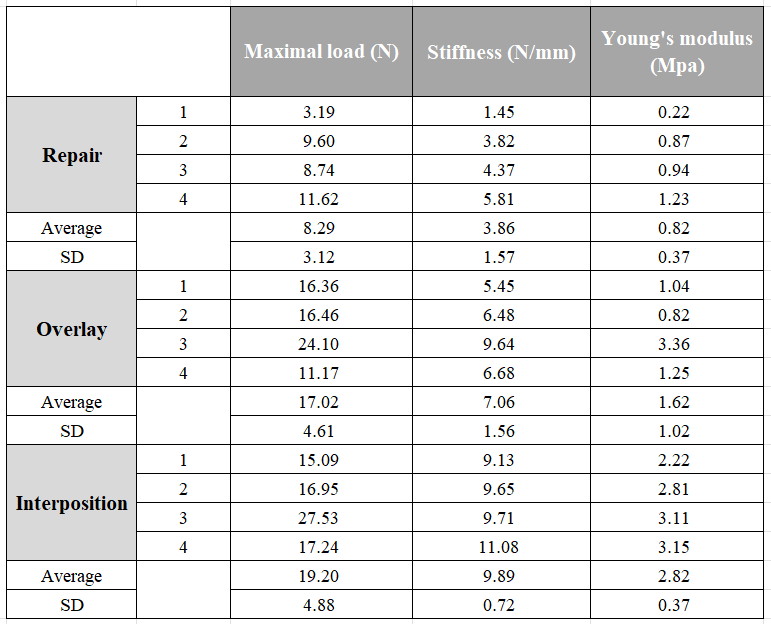

Supplement: S4 Table — The maximal load, stiffness, and Young’s modulus of the tendon-to-bone interface. (TIF) [file pone.0266030.s004.tif]
